# Somatic mutations in colorectal cancer are associated with the epigenetic modifications

**DOI:** 10.1111/jcmm.15799

**Published:** 2020-08-31

**Authors:** Hongwei Lei, Kaixiong Tao

**Affiliations:** ^1^ Department of Gastrointestinal Surgery Union Hospital Tongji Medical College Huazhong University of Science and Technology Wuhan China

**Keywords:** colorectal cancer, CRC, CTCF, H3K27me3, miRNA, somatic mutation

## Abstract

Colorectal cancer (CRC) mostly arises from progressive accumulation of somatic mutations within cells. Most commonly mutated genes like TP53, APC and KRAS can promote survival and proliferation of cancer cells. Although the molecular alterations and landscape of some specific mutations in CRC are well known, the presence of a somatic mutation signature related to genomic regions and epigenetic markers remain unclear. To find the signatures from a random distribution of somatic mutations in CRCs, we carried out enrichment analysis in different genomic regions and identified peaks of epigenetic markers. We validated that the mutation frequency in miRNA is dramatically higher than in flanking genomic regions. Moreover, we observed that somatic mutations in CRC and colon cancer cell lines are significantly enriched in CTCF binding sites. We also found these mutations are enriched for H3K27me3 in both normal sigmoid colon and colon cancer cell lines. Taken together, our findings suggest that there are some common somatic mutations signatures which provide new directions to study CRC.

## 1 INTRODUCTION

Early somatic mutations can cause developmental disorders, whereas the progressive accumulation of mutations throughout life can lead to cancers.^1^ Colorectal cancer (CRC) is one of most common malignancies in the world.^2^ It is estimated that approximately 145 600 new cases of CRC are diagnosed annually in the United States.^3^ Somatic mutations are becoming increasingly important biomarkers for cancer treatment decisions and outcome in patients with CRC. The altered DNA due to accumulated somatic mutations may act as a biologic driver of CRC. The location of somatic mutations, for example within specific somatic mutations of APC and TP53 (classical CRC somatic mutations genes), can influence biological processes involved in the development and progression of tumours, ultimately influencing the prognosis of CRC.^4^

With the development of next‐generation sequencing (NGS) technologies, sequencing throughput related to gene mutations has dramatically increased. The TCGA database for CRC somatic mutations can be used to study the distribution, differential expression and frequency of mutated genes. Including 6 classical CRC somatic mutations genes (TP53, APC, KRAS, FBXW7, PIK3CA and SMAD4),^5^, ^6^ novel somatic mutations, such as TCF7L2, TET2, TET3 and ERBB3, were also identified, which alluded to possible treatment avenues for CRC.^7^

NGS has revealed millions of somatic mutations associated with different human cancers,^8^ and the vast majority of them are located outside of coding sequences, making it challenging to directly interpret their functional effects,^9^ Further characterizing the somatic mutation landscape beyond protein‐coding regions will help distinguish tissue‐specific driver mutations in non‐coding regions. Examples of previous discoveries include recurrent mutations of the TERT promoter, which creates a binding motif for ETS transcription factors significantly increasing TERT transcriptional activity.^10^ In addition, somatic mutations in T‐cell acute lymphoblastic leukaemia introduce binding motifs for MYB, creating a super‐enhancer upstream of the TAL1 oncogene.^11^ In the non‐coding cancer genome, CCCTC binding factor (CTCF)/cohesin's binding sites (CBSs) are major mutational hotspots.^12^ Moreover, somatic mutations in miRNA exhibit potential role in tumorigenesis.^13^ Mutations in the miRNA coding regions will alter the expression of the target gene as the sequence of α‐miRNA is strictly complementary to the mature miRNA sequence.^14^

Here, we carried out enrichment analysis of somatic mutations in different genomic regions and analysed epigenetic marker peaks using 970 560 somatic mutations (covering 948 975 genome loci, chrM and other non‐canonical chromatin) from CRCs. The aim of this study was to survey the signature of somatic mutations in a diverse set of colorectal cancer genomes and obtain insights into the signature of somatic mutations in epigenetic markers of genomic regions.

## 2 MATERIALS AND METHODS

### 2.1 Data sources and collection

We included unbiased interpretation of somatic mutations from tumour sample dataset of Colon Adenocarcinoma (COAD) by harmonizing the results of seven algorithms, yielded by the uniform analysis of all The Cancer Genome Atlas (TCGA) exome data by the Multi‐Center Mutation‐Calling in Multiple Cancers (MC3) network (https://api.gdc.cancer.gov/data/1c8cfe5f‐e52d‐41ba‐94da‐f15ea1337efc).^15^ To reduce the false‐positive rate, we implemented two strategies to optimize driver detection and data quality. Briefly, we excluded hyper‐mutated tumours because of artefact sensitivity to high background mutation rates. All mutations that passed the MC3 filter criteria were included. Finally, we randomly selected 10 samples to do the following analysis by permutation test. Clinical information on TCGA was downloaded from the Genomic Data Commons Data Portal (https://portal.gdc.cancer.gov/). The data of histone modifications and chromatin assay come from Cistrome Data Browser.

### 2.2 RRBS sequencing data analysis

Reduced Representation Bisulfite Sequencing was download from GEO data set GSE95654. The raw paired‐end FASTQ reads were trimmed to remove both the adapter sequences and low‐quality bases. The alignment of bisulphite‐treated short reads to the reference genome hg19 was conducted as described by Cai et al.^16^ In brief, two read alignments were carried using the SOAP software to get the best hit for a given pair‐end short read. A straightforward seed‐and‐extension algorithm was then employed for the alignment, with two mismatches allowed in the seed (30 bp) and five mismatches in the whole read. Uniquely aligned reads that contained MspI digestion sites at their ends were retained for further analysis.^17^ Bisulphite conversion efficiency was calculated by using the C to T conversion rate for all cytosines in the CHH context (where H = A, T, or C). Even under the assumption that all 5mC in CHH nucleotides were products of conversion failure, the bisulphite conversion rate for each single‐base resolution approach was >99%, which ensured that the false‐positive rate was <1%.

### 2.3 ChIP‐seq sequencing data analysis

All ChIP‐seq sequencing data were mapped to the hg19 genome for human by using Bowtie2 (v2‐2.2.4)^18^ with parameters ‘‐q ‐‐phred33 ‐‐very‐sensitive ‐p 10’. Then, we removed duplicated reads for both pair‐end and single‐end data using SAMtools (v1.5).^19^ The bigwig files for IP/input ratio were generated from BAM files by using deepTools2 (v2.5.0)^20^ with command ‘bamCompare ‐b1 ChIP‐bam ‐b2 Input‐bam ‐‐ignoreDuplicates ‐‐minMappingQuality 30 ‐‐normalizeUsing RPKM ‐‐binSize 20 ‐‐smoothLength 60 ‐‐operation ratio ‐‐scaleFactorsMethod None ‐p 20’. BAM files of mapping results were merged for the same sample using SAMtools and converted to BED format by using BEDTools.^21^ Peaks of regulatory regions were called for each sample by using MACS (v1.4.2)^22^ from bed files of ChIP‐seq with parameters ‘‐w ‐S ‐p 0.00001 ‐g mm’. The input signal was used as the control to call peaks for the ChIP‐seq data set. The heatmap plot of signals centred on peaks was implicated by deepTools2 subcommand plotHeatmap. Annotation of peaks to nearest genes and genomic regions (eg promoters, CG‐rich regions, repeat regions) was performed with annotatePeaks.pl (default settings) in HOMER (v4.91).^23^

### 2.4 Somatic mutation annotations

Somatic mutations were annotated and analysed by ANNOVAR v2018Apr16,^24^ including annotations of population allele frequencies from the Exome Aggregation Consortium (ExAC v0.3), status in dbSNP (v147) and predictions of functional effects by MutationTaster, PolyPhen2, SIFT and CADD v1.3. Synonymous SNVs (single nucleotide variants), in‐frame indels, as well as variants, predicted to have non‐deleterious functional effects or population allele frequencies greater than 10% were not reported. Mann‐Whitney U test was used to calculate the mean mutation rate of International Cancer Genome Consortium (ICGC) and TCGA databases in miRNA regions.

### 2.5 Motif discovery

De novo motifs were calculated with the HOMER findMotifsGenome.pl command with default parameters. Enrichment of de novo motifs was calculated using the findKnownMotifs.pl program in HOMER with default parameters.

### 2.6 CpG OE calculation

CpG_O/E_ (observed/expected for CpG) was defined as the ratio of the actual CpG density which represent the composition of nucleotide. CpG_O/E_ was calculated as follows:

$$\text{CpG}\frac{O}{E}=\frac{\text{Number of CpG}}{\text{Number of C}\times \text{number of G}}\frac{N^{2}}{N‐1}$$

where N is the size of the sequence segment (window) in which total nucleotides were analysed.^25^ A 400 bp window (N = 400) moving across the sequence at 1 bp intervals was chosen to monitor the characteristic variations.

## 3 RESULTS

### 3.1 Somatic mutations are enriched in miRNA regions

**Figure 1:**
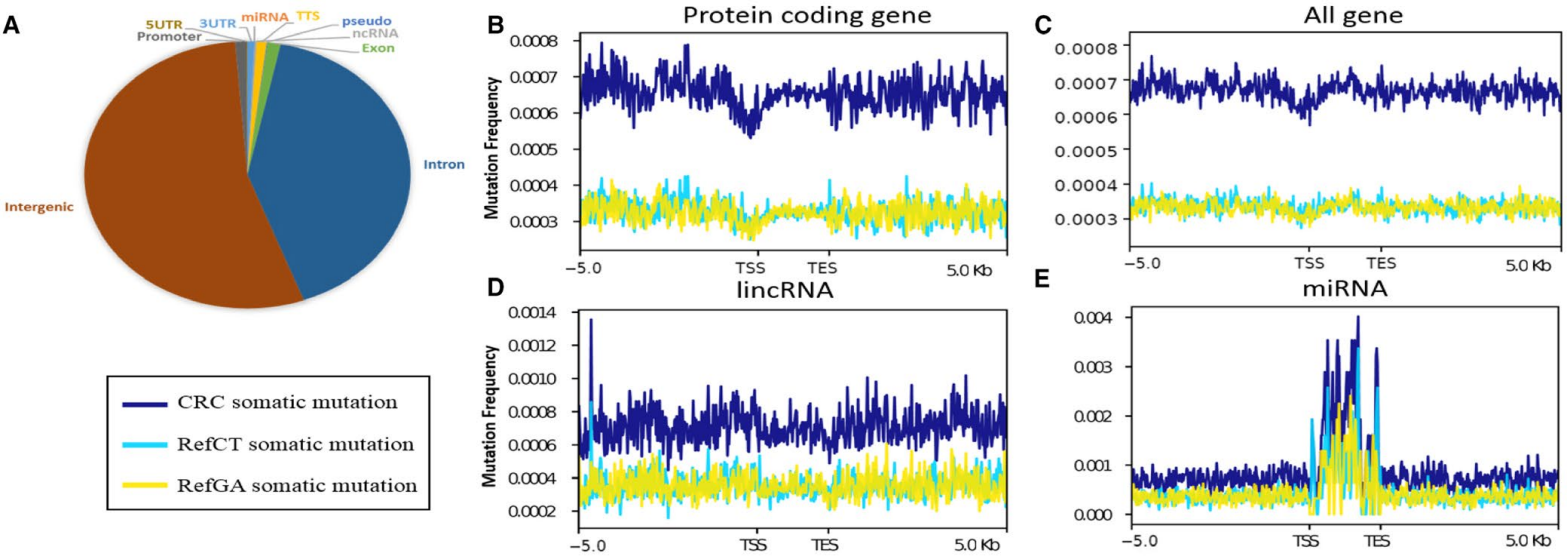
Genomic distribution of somatic mutations. A, Distribution of somatic mutations in basic gene elements indicates that somatic mutations mostly located in intergenic and intronic region. B‐E, Distribution of somatic mutations in upstream 5k bp, body (normalized into 2k bp) and downstream 5k bp of (B): protein‐coding genes, (C): all genes, (D): lncRNA and (E): miRNA. Ref‐CT (light green), Ref‐GA (yellow) and CRC (blue) somatic mutations represent C/T→G/A, G/A→C/T and all of the somatic mutations, respectively. ‘TSS’ indicates the transcriptional starting sites while ‘TES’ indicates the transcriptional end sites

**Figure 2:**
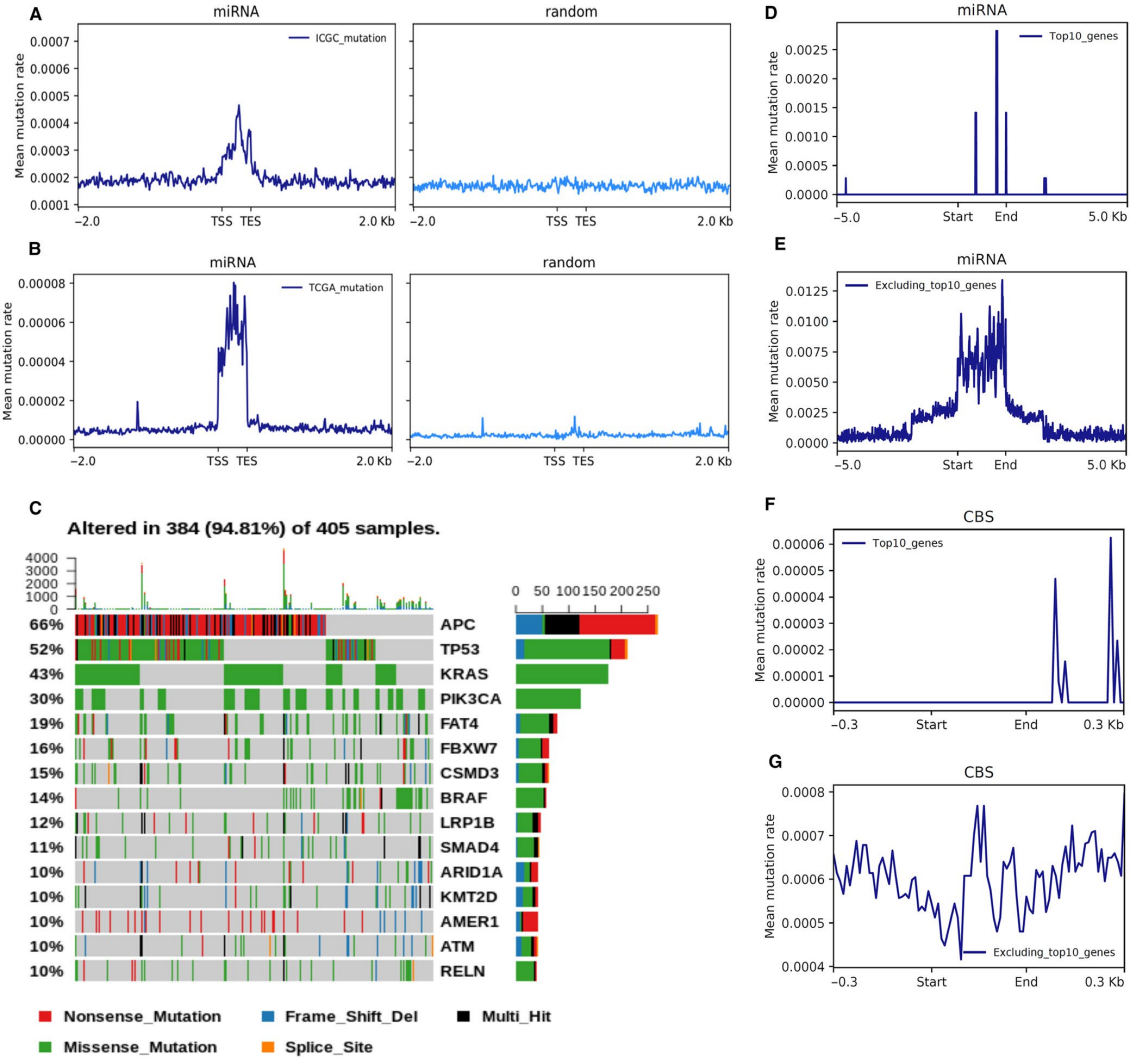
Somatic mutations are enriched in miRNA regions but not random regions. A, Mean mutation rate in ICGC (International Cancer Genome Consortium) database of miRNA (left panel) and random regions (right panel): Mann‐Whitney U Test, *P* = 1.63e−9; B, Mean mutation rate in TCGA (The Cancer Genome Atlas) database of miRNA (left panel) and random regions (right panel): Mann‐Whitney U Test, *P* < 2.2e−16. ‘TSS’ indicates the transcriptional starting sites while ‘TES’ indicates the transcriptional end sites. C, Waterfall plots for highly mutated CRC genes (more than 10% mutation rate) based on 405 samples. Top 10 key CRC genes include APC, TP53, KRAS, PIK3CA, FAT4, FBXW7, CSMD3, BRAF, LRP1B and SMAD4. D‐G, Mutation distribution of (D): top 10 key CRC genes in miRNA regions, (E): excluding top 10 key CRC genes in miRNA regions, (F): top 10 key CRC genes in CBS regions, (G): excluding top 10 key CRC genes in CBS regions

To explore the distribution of somatic mutations, we examined the mutation regions in Homer v4.91 by mutation annotation. We annotated 528 087; 398 899; 12 613; 10 890; 9752; 6854; 1931; 814; 690; and 29 somatic mutations located in intergenic, intronic, exonic, promoter, TTS, 3’UTR, ncRNA, 5’UTR, pseudo‐gene and miRNA regions, respectively. We analysed the distribution of mutation frequency around the region body and 5’ flanking regions. We observed that the mutation frequency was much lower in the promoter of protein‐coding genes than within the gene body (Figure 1A‐B). The decline of mutation frequency in promoters was not apparent in all genes (Figure 1C) or non‐coding genes such as lncRNA genes (Figure 1D). Interestingly, we found that the mutation frequency in miRNA regions was dramatically higher than in the flanking regions (Wilcoxon *P* = 1.46 × 10^−12^) (Figure 1E), which was not observed in other subsets of genes. The mutation sites were enriched in the miRNA regions than in random regions with *P* = 1.63e−9 and *P* < 2.2e−16, respectively (Figure 2A‐B).

To explore whether the mutations of key CRC genes (ie APC, TP53 and KRAS) affect the differential enrichment of somatic mutations in different regions, we performed waterfall plots for top 30 mutated genes in CRC and found the top 10 genes were mutated in more than 10% of CRC tumours. These highly mutated CRC genes include APC, TP53, KRAS, PIK3CA, FAT4, FBXW7, CSMD3, BRAF, LRP1B and SMAD4 (Figure 2C). Then, we separated somatic mutations into two groups with or without top 10 key CRC genes and performed the composite analysis. Finally, we found the somatic mutations were enriched in both key CRC genes and non‐key CRC genes (Figure 2D‐E). However, we did not observe enrichment of mutations in CBS regions for these key CRC genes while mutations were enriched in non‐key CRC genes (Figure 2F‐G). Together, these results indicate that mutations of key CRC genes (ie APC, TP53 and KRAS) do not affect the enrichment of somatic mutations in different regions.

We next examined whether mutations accumulated at miRNA regions in other cancers. We found a similar miRNA signatures in 8 different cancer subtypes: Breast cancer (BRCA), cholangiocarcinoma (CHOL), oesophageal carcinoma (ESCA), cervical squamous cell carcinoma (CESC), uterine cancer (UCEC), stomach and oesophageal carcinoma (STES), lung adenocarcinoma (LUAD) and sarcoma (SARC) (Figure S1). We tried the different signatures of mutational processes, but we did not find any significant differences of mutations patterns. LAML (acute myeloid leukaemia), LGG (Brain Lower Grade Glioma), ORCA (oral carcinoma), LICA (liver carcinoma), KIRC (Kidney renal clear cell carcinoma), GACA (gastric cancer), BLCA (Bladder Urothelial Carcinoma) cancer from TCGA project showed enrichment of mutations in miRNA regions while GACA and ESAD (oesophageal adenocarcinoma) showed mutational enrichment in CBS regions (Figure S2). Moreover, LAML, LGG, GACA, KIRC, LICA, PRAD (Prostate Adenocarcinoma) and UCEC cancers from ICGC project also showed enrichment of mutations in miRNA regions. And we confirmed that GACA and ESAD showed mutational enrichment in CBS regions (Figure S3). Both SNPs (single nucleotide polymorphisms) and InDels (insertions and deletions) would affect the patterns of mutations although the proportion of small InDels account for only about 8% of all somatic SNVs (single nucleotide variants). Compared with that in lncRNA and random regions, we did observe significant enrichment of mutations in CBS binding regions as well as miRNA regions for both SNPs and InDels (Figure S4). Therefore, our observations supported that not only SNPs but also InDels contribute to the specific patterns in the genomic and CBS regions.

In addition, we classified 2374 miRNAs into three categories: 1307 miRNAs overlapped with introns of protein‐coding genes (intron miRNA), 156 miRNAs overlapped with CDS regions (CDS miRNAs) and 911 miRNAs located in intergenic regions (intergenic miRNAs). Interestingly, we found mutations are enriched in intergenic miRNAs but not in CDS miRNAs compared flanking regions (Figure S5A‐B). Moreover, we observed mutational enrichment in both promoters and bodies of intron miRNAs while there is no enrichment of mutations in randomly selected regions (Figure S5C‐D).

### 3.2 Somatic mutations are enriched in CTCF binding sites

**Figure 3:**
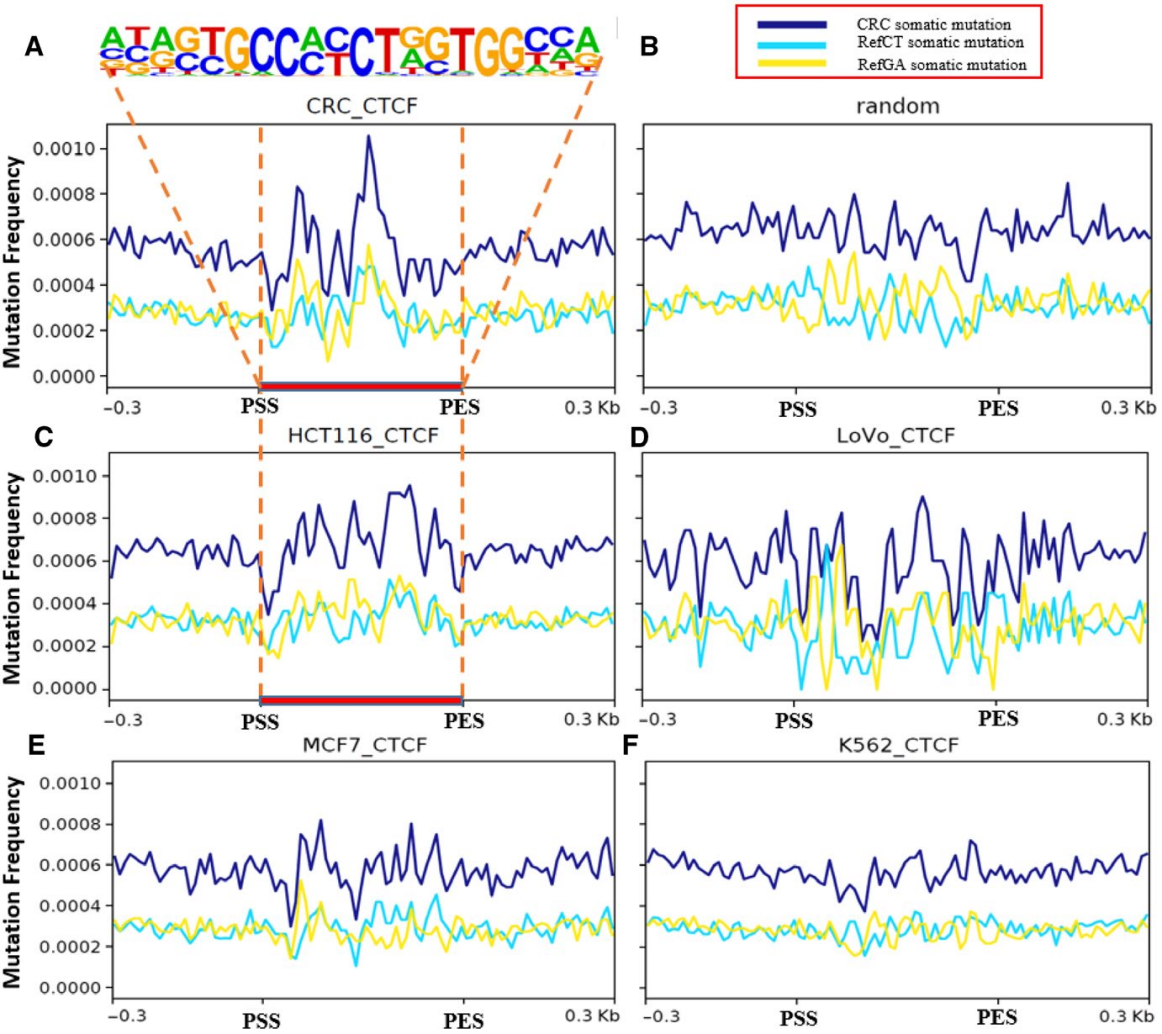
Distribution of somatic mutations in and ±300 bp of CTCF binding sites. A, Mutation frequency of somatic mutations and motif enrichment surrounding CTCF binding sites from CRC patients. B, Mutation frequency of somatic mutations in random regions corresponding to CTCF binding sites from CRC patients. C‐F, Mutation frequency of somatic mutations in CTCF binding sites from cell lines (C): HCT116, (D): LoVo (E): MCF7 and (F): K562. Ref‐CT (light green), Ref‐GA (yellow) and CRC (blue) somatic mutations represent C/T→G/A, G/A→C/T and all of the somatic mutations, respectively. ‘PSS’ indicates the peak's starting sites while PES means peak's end sites

Given genomic CTCF/cohesin‐binding sites (CBSs) are frequently mutated hotspots in numerous malignancies,^12^ we performed analyses of mutation clusters in genomic regions by MACS2 with a q‐value cutoff of < 0.05. We confirmed that somatic mutations were significantly enriched in CBSs in patients of CRC (31,252 sites with Wilcoxon *P* = 3.18 × 10^−15^) (Figure 3A) while there was no enrichment in random regions (Figure 3B) compared with 600 flanking regions. Moreover, we observed mutation hotspots in the CTCF binding regions of CRC cell line HCT116 (Wilcoxon *P* = 5.42 × 10^−9^) (Figure 3C) but not of other cancer cell lines, such as MCF7 and K562 (Figure 3D‐F). The CTCF peaks of HCT116, LoVo, MCF7 and K562 were derived from public datasets deposited in GEO database.

### 3.3 Somatic mutations are correlated with low CpG_O/E_ value and high‐CpG methylation

**Figure 4:**
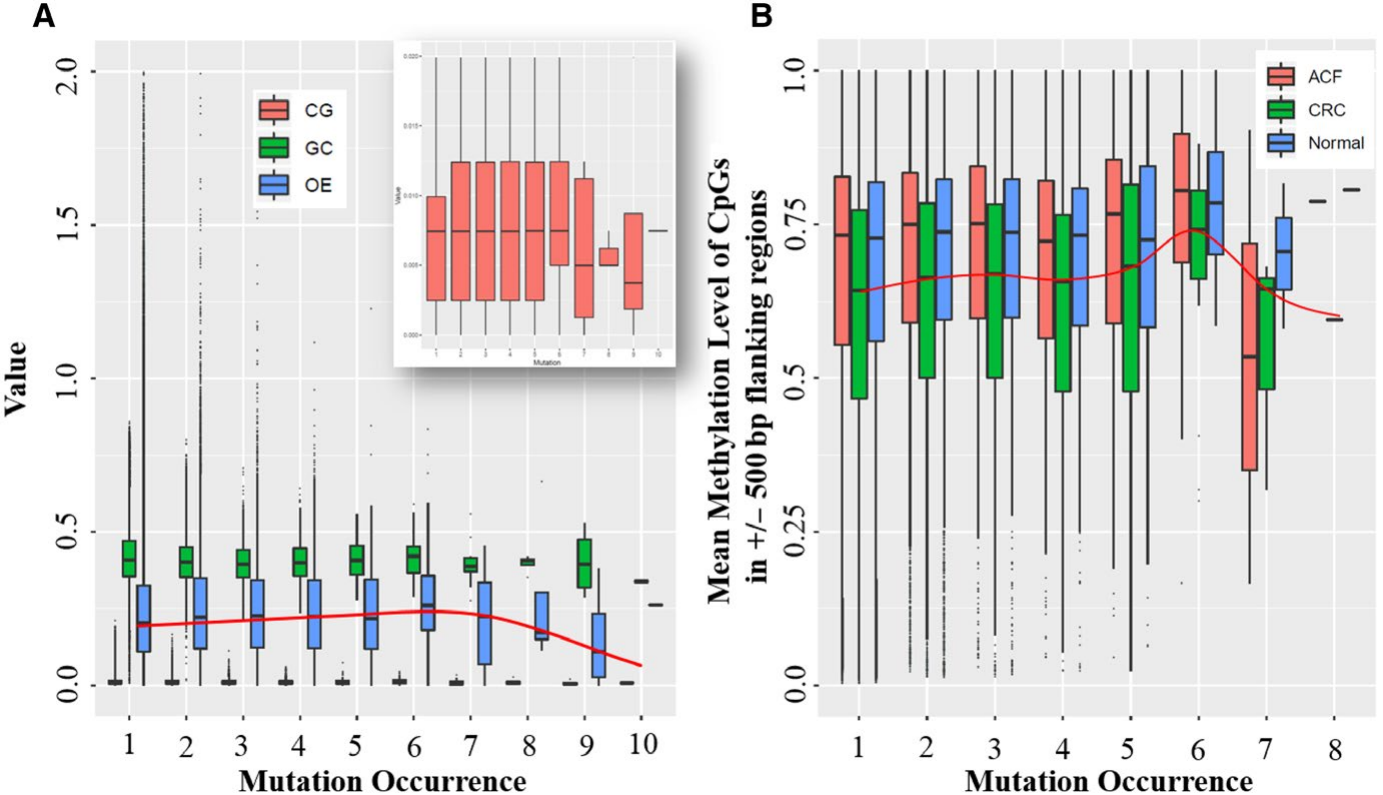
Relationship between somatic mutation and CpG density or CpG methylation. A, Mutation occurrences in CpG content (CG), GC content (GC) and CpG_O/E_ (ratio of the actual CpG density) show that mutation frequency is negatively correlated with OE rather than CG or GC. B, Mean methylation level of CpGs in ACF (human aberrant crypt foci), CRC and normal samples reveal that CRC methylation is much lower than in normal adjacent tissues or ACF samples

A high rate of CpG mutations should deplete the frequency of CpG sites so that CpG_[O/E]_ decreases.^26^ Therefore, we investigated the relationship between mutation occurrence and CpG content (CG), GC content (GC) and CpG[O/E] in 400 bp centred on each mutation.^16^ This revealed the mutation frequency was significantly negatively correlated with OE rather than CG or GC (Figure 4A). Methylated CpG dinucleotides can lead to 10‐fold higher C→T mutation rate than at unmethylated sites,^25^, ^27^ less is known about whether and how the methylation level alters the mutation rate, in particular, at single‐base resolution. Here, we calculated the mean CpG methylation level of 1k regions centred on each somatic mutation. We found methylation versus the mutation occurrence revealed CRC methylation was much lower than in normal adjacent tissues or human aberrant crypt foci (ACF) samples (Figure 4B). However, the mean methylation was slightly elevated when the value of mutation occurrence is under 6 (Figure 4B). Methylation was detected by RRBS (GEO data set GSE95654), which covered high‐CpG islands and promoters, requiring additional validation via genome‐wide MethylC‐seq in CRC.

### 3.4 Somatic mutations are enriched in poised enhancers marked by H3K27me3

**Figure 5:**
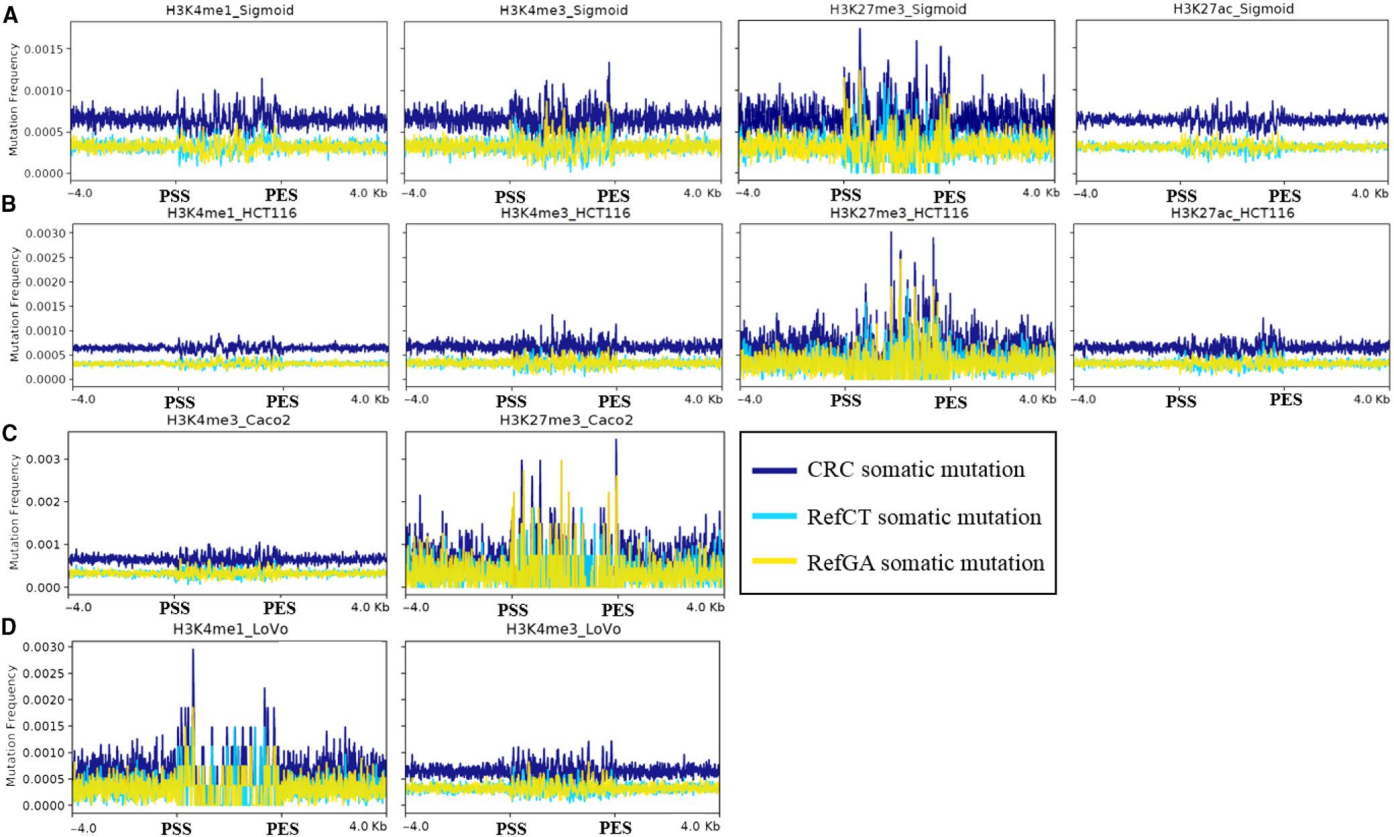
Distribution of somatic mutations in and ±4 kbps of peaks of histone modifications. A, Distribution of somatic mutations in peaks of histone modifications H3K4me1, H3K4me3, H3K27me3 and H3K27ac in sigmoid colon. B, Distribution of somatic mutations in peaks of histone modifications H3K4me1, H3K4me3, H3K27me3 and H3K27ac in HCT116 cell line. C, Distribution of somatic mutations in peaks of histone modifications H3K4me3 and H3K27me3 in Caco2 cell line. D, Distribution of somatic mutations in peaks of histone modifications H3K4me1 and H3K4me3 in LoVo cell line. Ref‐CT (light green), Ref‐GA (yellow) and CRC (blue) somatic mutations represent C/T→G/A, G/A→C/T and all of the somatic mutations, respectively. ‘PSS’ indicates the peak's starting sites while PES means peak's end sites

By analysing H3K4me1, H3K4me3, H3K27me3 and H3K27ac in sigmoid colon, HCT116, Caco2 and LoVo cell lines, we plotted the distribution of somatic mutations in/around the peaks of these histone modifications. We found that somatic mutations were enriched in peaks of H3K27me3 in both normal sigmoid colon (Wilcoxon *P* = 6.40 × 10^−10^) and colon cancer cell lines HCT116 (Wilcoxon *P* = 3.96 × 10^−14^) and Caco2 (Wilcoxon *P* = 5.06 × 10^−13^). However, the somatic mutations were not enriched in peaks of H3K4me1, H3K4me3 and H3K27ac (Figure 5). As H3K27me3 demarcates poised enhancers, we propose that poised enhancers marked by H3K27me3 are frequently mutated in human colorectal cancers.^28^ This phenotype is consistent with our observation that mutation rate declines in the promoter regions of protein‐coding genes (Figure 1B). In addition, we also found that the mutation rate declines in body regions of super enhancers (from SEA database, https://academic.oup.com/nar/article/48/D1/D198/5610346) compared with flanking regions while the mutation rate increases in body regions of predicted general enhancers (from JEME database, https://www.nature.com/articles/ng.3950) compared with flanking regions (Figure S6).

### 3.5 Somatic mutations are oscillated in chromatin open regions

**Figure 6:**
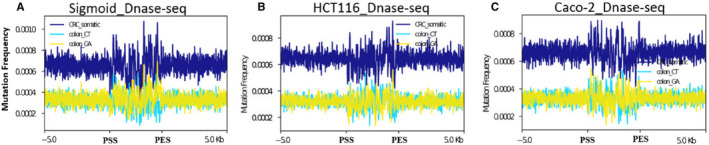
Distribution of somatic mutations in and ±5 kbps of peaks of DNase‐seq in sigmoid colon. A, Sigmoid; B, HCT116 and (C) Caco‐2. colon_CT (light green), colon_GA (yellow) and CRC (blue) somatic mutations represent C/T→G/A, G/A → C/T and all of the somatic mutations, respectively. ‘PSS’ indicates the peak's starting sites while PES means peak's end sites

Chromatin organization contributes to regional variation in mutation rate, but differently among mutation types. In both germline mutations and somatic mutations, base substitutions are more abundant in regions of closed chromatin, perhaps reflecting error accumulation late in replication.^29^ In contrast, a distinctive mutational state with very high levels of insertion or deletions (indels) and substitutions is enriched in regions of open chromatin.^29^ In our study, we found regions of open chromatin show alternately higher and lower mutation frequency, compared to flanking regions (Figure 6). We define this mutation distribution pattern as oscillation. Consistently, we found somatic mutation fluctuated in flanking regions between normal sigmoid colon and colon tumour cell lines HCT116 and Caco‐2 (Figure 6).

## 4 DISCUSSION

In this paper, we have shown that somatic mutations are enriched in the regions of miRNA which functions in RNA silencing and post‐translational regulation of gene expression. Any mutations in the miRNA coding region can modify target gene expression and may alter binding leading to the activation of various biological and pathological processes, including expression of tumour suppressor genes and oncogenes.^30^ For example, expression of E2F1 in colon cancer was increased fourfold after the somatic mutation of miR136‐5p compared with normal tissue.^31^ Finding somatic mutations of miRNA in colon cancer may help identify new therapeutic targets for CRC treatment.

Here, we confirmed that in CRC, mutation hotspots enriched at CBSs that disrupt CTCF binding, consistent with previous reports in gastrointestinal cancers (GC).^12^ CTCF is a DNA‐binding protein essential for the maintenance of genome architecture by mediating inter‐chromosomal contacts.^32^ Somatic mutations of CBSs may disrupt the CTCF binding leading to dysregulation of gene expression. Compared with gastrointestinal cancers, 25% of all gastric tumours are mutated in at least one of the 11 CBS hotspots.^12^ We observed a high frequency of mutation hotspots in the CTCF binding regions of CRC and HCT116, compared to MCF7 or K562. In addition, in GC, microsatellite instability mutation profiles showed a positive association with heterochromatin and repressive domains. Here, we first verified the relationship between the somatic mutations and histone modifications in CRC by comparing different epigenetic markers between CRC and normal tissues.

Epigenetic modifications, such as histone methylation and acetylation, can act as regulatory switches for gene transcription, and their dysfunction can give rise to developmental abnormalities^33^ and carcinogenesis.^34^ Previous studies have focused exclusively on the effects of cancer‐associated mutations on histones themselves,^35^ but little is known about the relationship between the somatic mutations and histone modifications. Here, in this study, we found that somatic mutations showed no enrichment in regions of open chromatin or histone marks of active promoters (H3K4me3) or enhancers (H3K27ac) but exhibited strong relationship with poised enhancers marked by H3K27me3, linked to gene repression. Previous studies have illustrated that nucleosome positioning is considered essential by affecting the mutability of genomic sequences and the rate of base substitution mutations.^36^, ^37^ In human, the mutation rates of T‐>C, A‐>G, G‐>T, C‐>A, T‐>A and A‐>T were promoted and correlated with certain histone modifications in nucleosome‐occupied regions.^38^ High mutation density of repressive histone mark‐associated regions has been reported in previous research.^39^ Comparing to active enhancers, poised enhancers may have limited accessibility to DNA repair complexes. In addition, active enhancers give rise to eRNAs,^40^ which play active roles in transcriptional regulation.^41^ When somatic mutations occur in active enhancers, they are unlikely to be accumulated due to the aberrant transcription. Relative to active enhancers, poised enhancers do not give rise to eRNAs. Thus, somatic mutations in poised enhancers can be possibly enriched. However, whether these mutational signatures or differential peak enrichment for H3K27me3 in normal versus cancerous tissues exists in other human cancers remains unknown.

The formal definition of CpG islands is a region with at least 200 bp, a GC percentage greater than 50%, and CpG_O/E_ greater than 60%.^42^ This CpG content will change as somatic mutation of CpG islands rates increase. We validated that in CRC, mutation frequency was negatively correlated with CpG_O/E_ value rather than CpG content or GC content. Moreover, we found that the average value of CpG_O/E_ was much lower than in normal colon tissue, which meant the somatic mutation frequency CRC CpG islands was higher than normal colon tissue. Methylated cytosine within a gene can alter its expression levels.^43^ In mammals, almost 80% of CpG cytosines are methylated.^44^ However, we found that this methylation level was decreased in CRC compared with normal adjacent tissues, which suggested that somatic mutations and methylation of CpG islands may have an impact on CRC tumorigenesis. It is worth noting that allele‐specific mutations and genomic imprinting are currently hot topics in research community. We tried to explore whether somatic mutations are enriched on the same allele or different allele of regions marked by high‐CpG methylation and H3K27me3. However, we could not have the access to the raw sequencing data from TCGA or ICGC projects and failed to apply the authority of raw data deposited in dbGaP database. We hope we could elucidate their relationships for further research.

Somatic mutations are a major source of CRC development. Recent developments in high‐throughput sequencing have made mutation detection easier. Our study highlights the use of large‐scale sequencing data as a bioinformatic strategy for establishing relevant somatic mutations underlying the biological effects of CRC. To our knowledge, this is the first report exploring signatures of somatic mutations in both genomic regions (miRNA) and epigenetic markers (H3K27me3) in CRC. Since large‐scale of gene annotations can be readily accessed to find epigenetic signatures, further attention on somatic mutation in CRC may help reveal new therapeutic targets for CRC treatment.

## CONFLICT OF INTEREST

The authors declare that they have no competing interests.

## AUTHOR CONTRIBUTION

**Hongwei Lei:** Formal analysis (equal); Methodology (equal); Software (equal); Writing‐original draft (equal). **Kaixiong Tao:** Funding acquisition (equal); Supervision (equal); Writing‐review & editing (equal).

## Supporting information

Supplementary Material

## Data Availability

Reduced Representation Bisulfite Sequencing was download from GEO data set GSE95654.
