# Supplementary material for: Somatic mutations in colorectal cancer are associated with the epigenetic modifications
Source: J Cell Mol Med. 2020 Aug 31;24(20):11828–36. doi: 10.1111/jcmm.15799 (PMC7579689; doi:10.1111/jcmm.15799)
Supplement: Supplementary file 1 — Supplementary Material [file JCMM-24-11828-s001.docx]

**Supplementary**


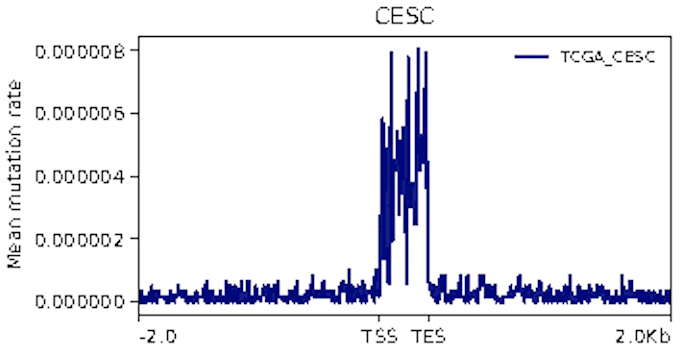

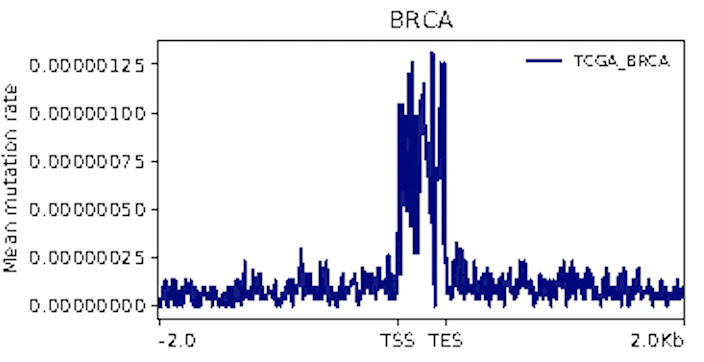

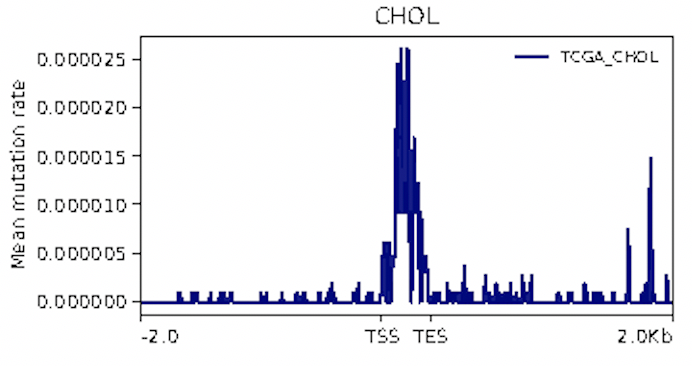

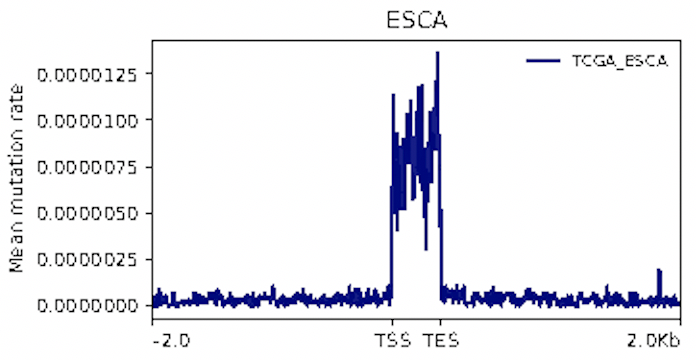

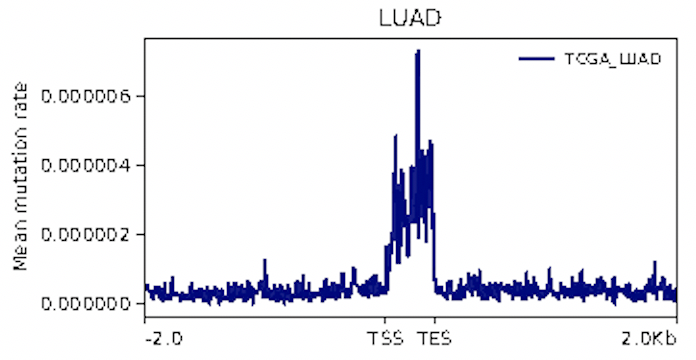

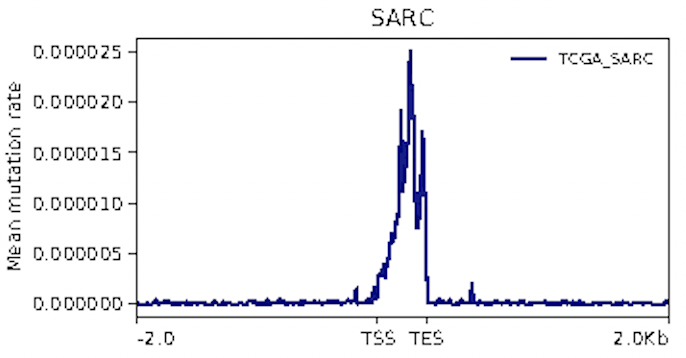

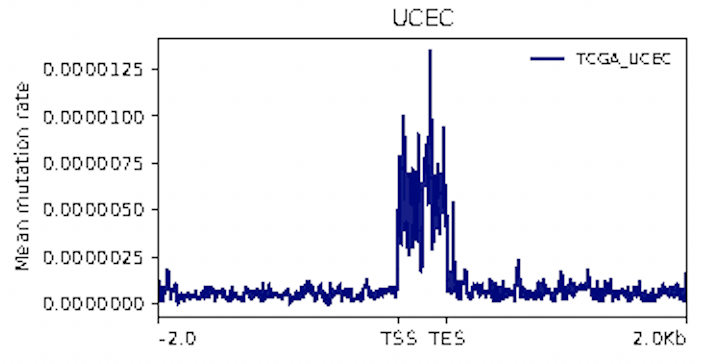

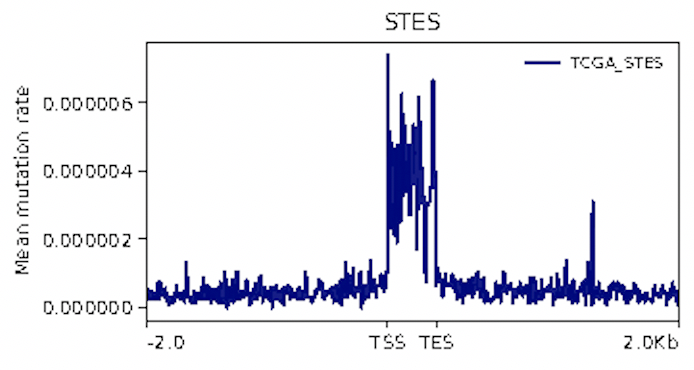


**Fig. S1.** miRNA regions mutation in other 8 types of cancer based on TCGA database. Abbreviation: Breast cancer (BRCA), cervical squamous cell carcinoma (CESC), lung adenocarcinoma (LUAD), cholangiocarcinoma (CHOL), uterine cancer (UCEC), sarcoma (SARC), esophageal carcinoma (ESCA), stomach and esophageal carcinoma (STES).


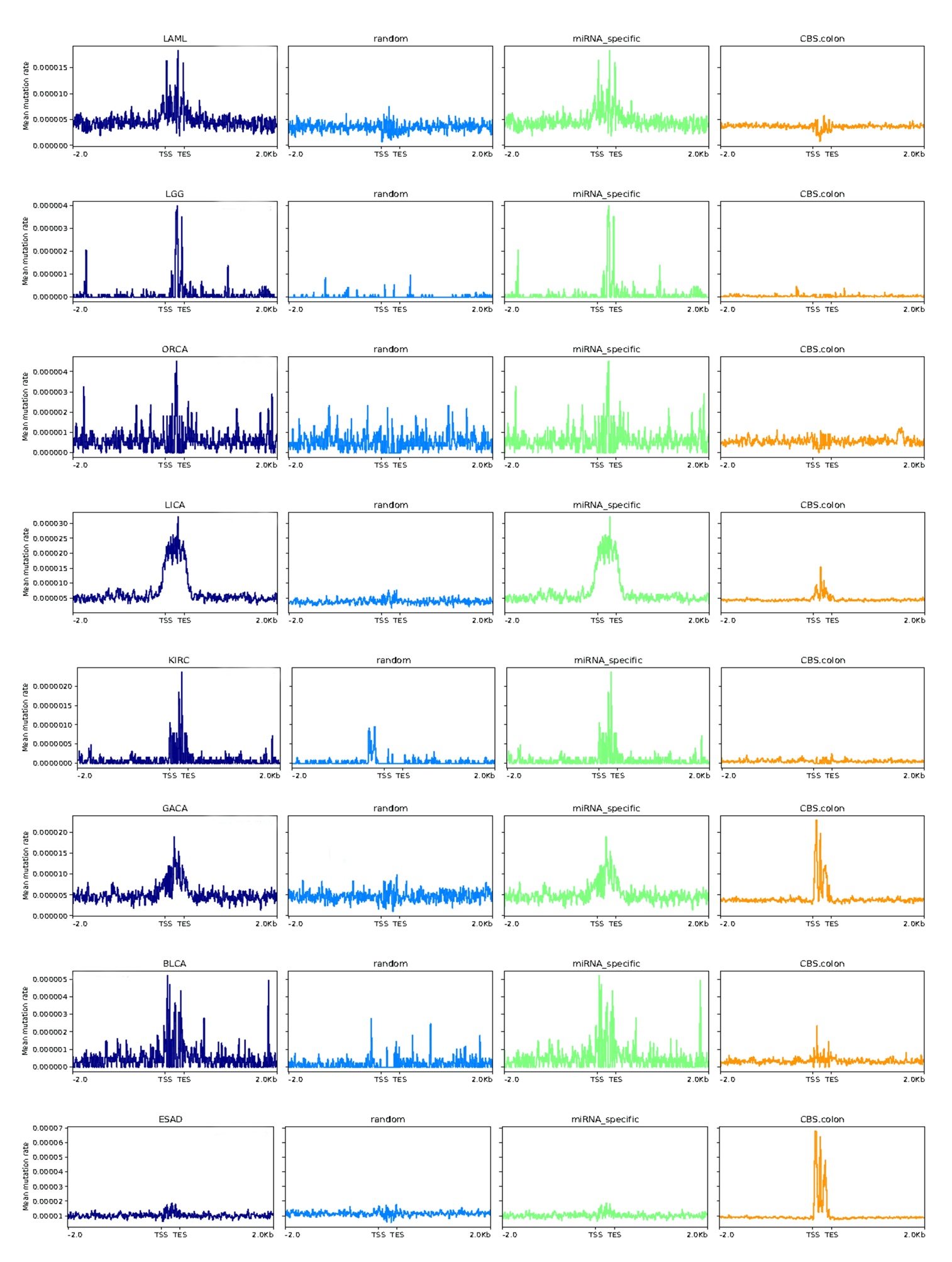


**Fig. S2.** Mutational signatures from TCGA project in 8 different cancers. Abbreviation: LAML (Acute Myeloid Leukemia), LGG (Brain Lower Grade Glioma), ORCA (oral carcinoma), LICA (liver carcinoma), KIRC (Kidney renal clear cell carcinoma), GACA (gastric cancer), BLCA (Bladder Urothelial Carcinoma) and ESAD (esophageal adenocarcinoma).

**
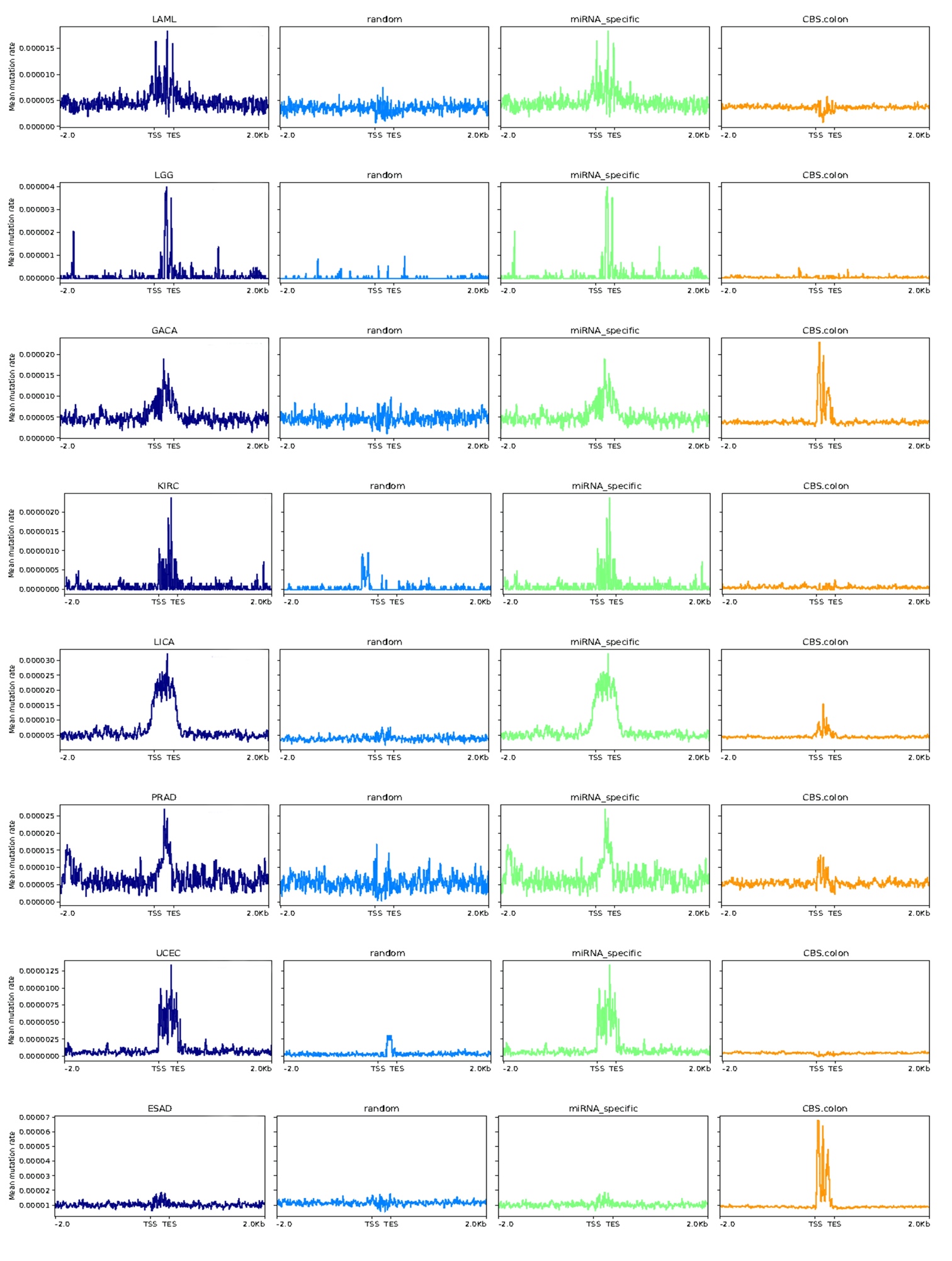
**

**Fig. S3.** Mutational signatures from ICGC project in 8 different cancers. Abbreviation: LAML (Acute Myeloid Leukemia), LGG (Brain Lower Grade Glioma), GACA (gastric cancer), KIRC (Kidney renal clear cell carcinoma), LICA (liver carcinoma), PRAD (Prostate Adenocarcinoma), UCEC (uterine cancer) and ESAD (esophagea l adenocarcinoma).

**
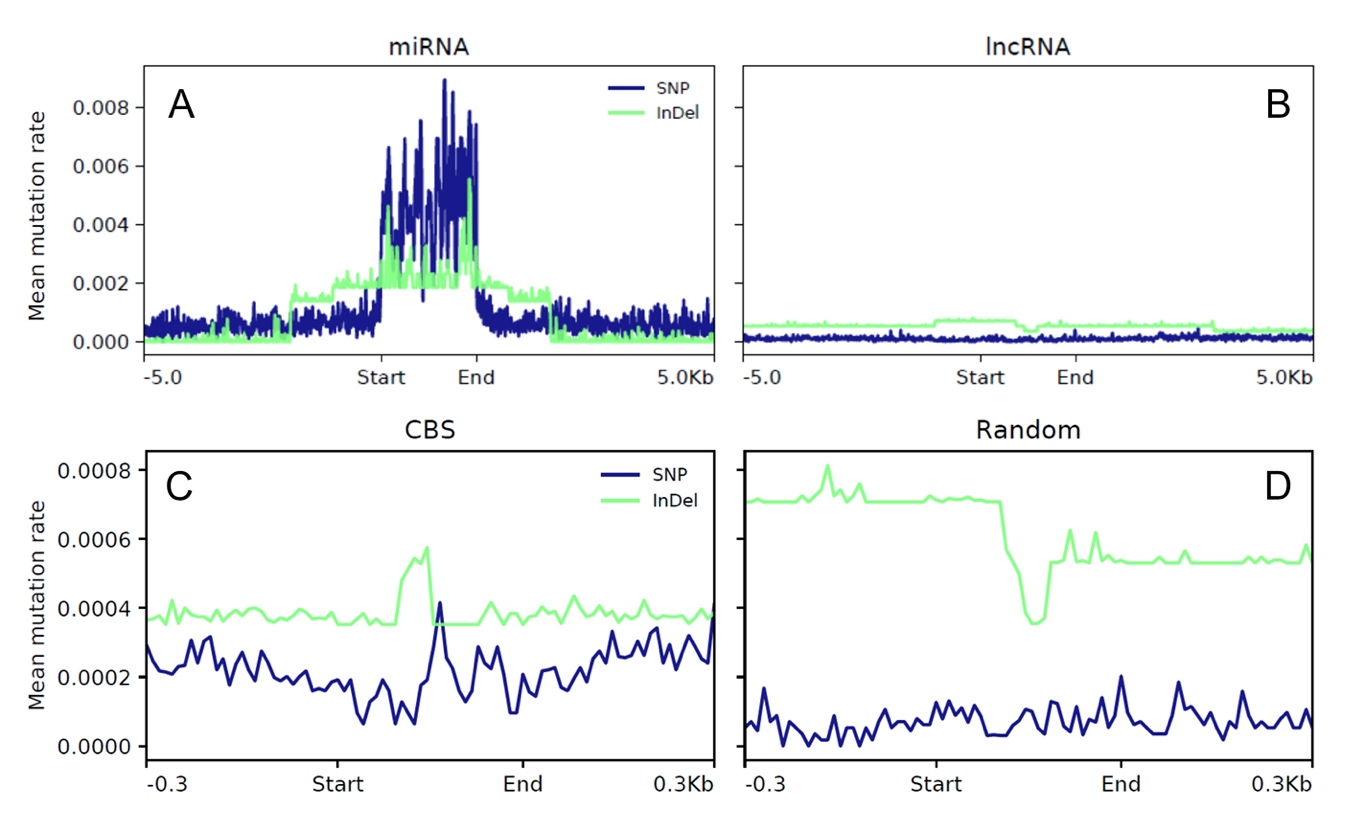
**

**Fig. S4.** SNP (single‐nucleotide polymorphism) and InDel (insertions and deletion) mutation rate in different genomic regions (A): miRNA, (B): lncRNA, (C): CBS, (D): random regions.


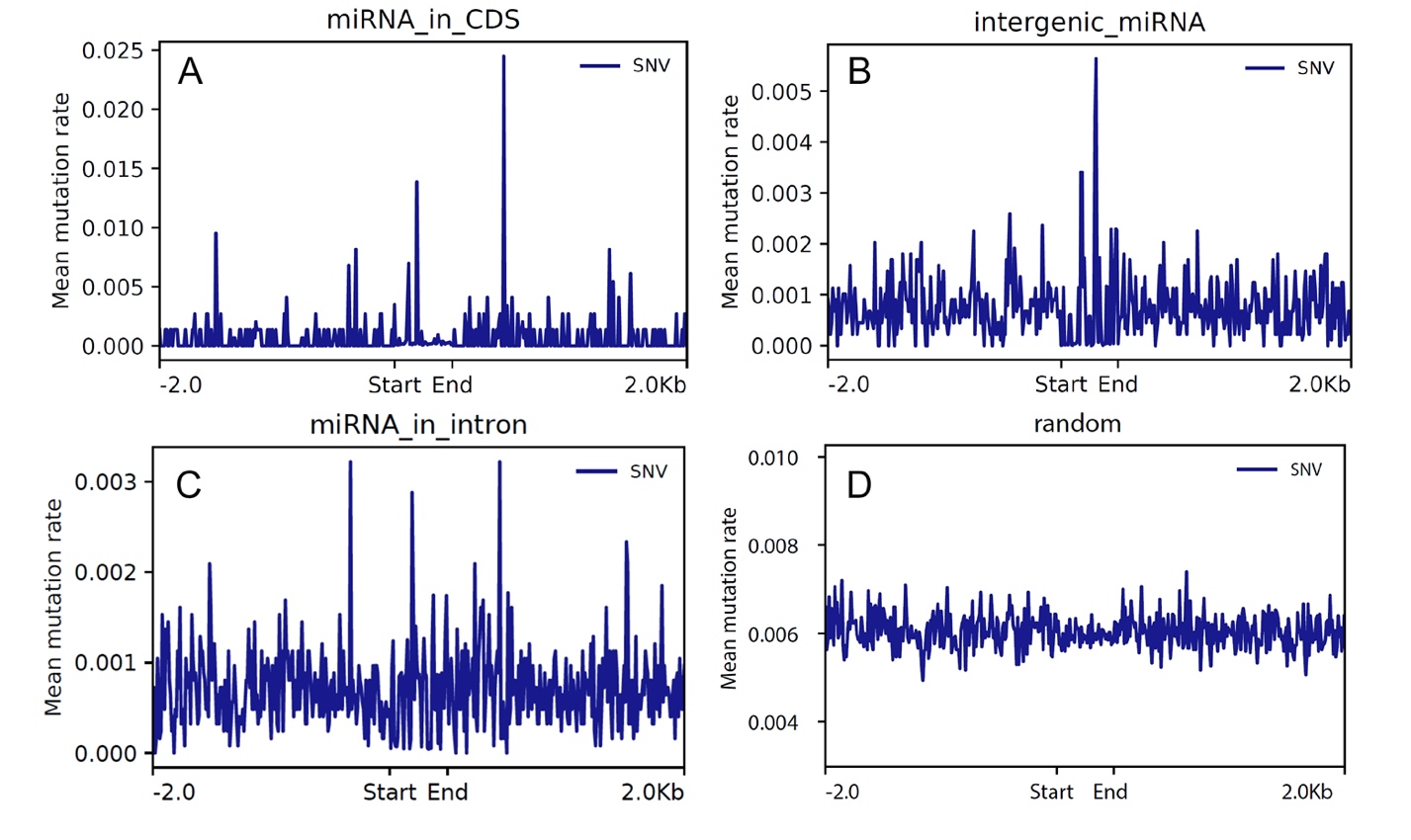


**Fig. S5.** SNV mutation rate in (A): CDS miRNA, (B): intergenic miRNAs, (C): intron miRNA and (D) random regions.

**
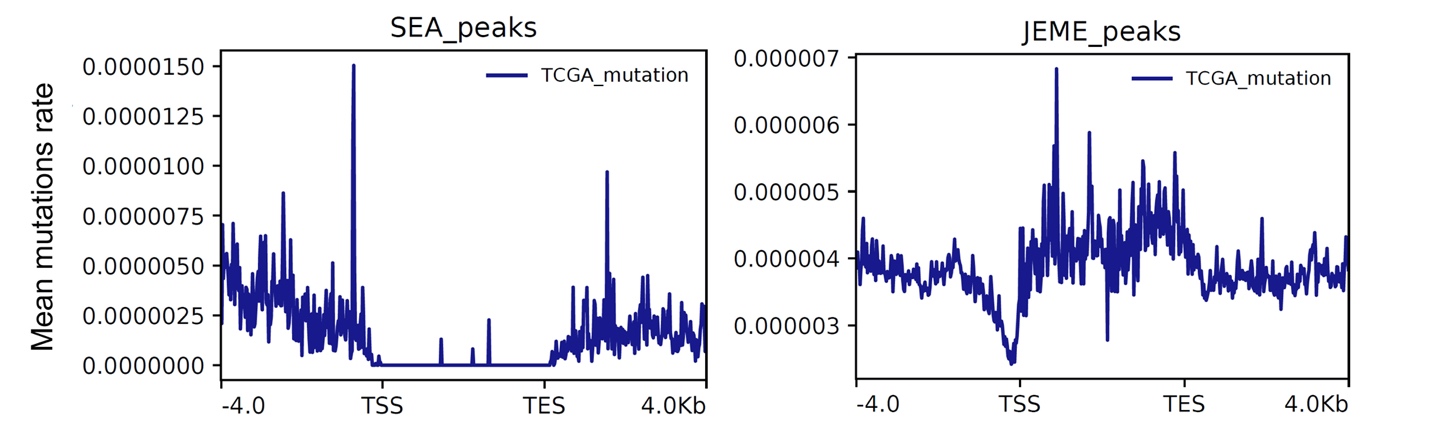
**

**Fig S6.** The mutation rate declines in body regions of super enhancers (from SEA database, left panel) and increases in body regions of predicted general enhancers (from JEME database, right panel) compared with flanking regions.
